# Validation of the General Medication Adherence Scale in Saudi Patients With Chronic Diseases

**DOI:** 10.3389/fphar.2019.00633

**Published:** 2019-06-04

**Authors:** Atta Abbas Naqvi, Dhafer Mahdi AlShayban, Syed Azizullah Ghori, Mansour Adam Mahmoud, Abdul Haseeb, Hani Saleh Faidah, Mohamed Azmi Hassali

**Affiliations:** ^1^Department of Pharmacy Practice, College of Clinical Pharmacy, Imam Abdulrahman Bin Faisal University, Dammam, Saudi Arabia; ^2^Department of Clinical and Hospital Pharmacy, College of Pharmacy, Taibah University, Medina, Saudi Arabia; ^3^Department of Pharmacy Practice, College of Pharmacy, Umm Al Qura University, Makkah, Saudi Arabia; ^4^Department of Microbiology, Faculty of Medicine, Umm Al Qura University, Makkah, Saudi Arabia; ^5^Discipline of Social and Administrative Pharmacy, School of Pharmaceutical Sciences, Universiti Sains Malaysia, Penang, Malaysia

**Keywords:** medication adherence, patient compliance, medication persistence, chronic illness, Saudi Arabia

## Abstract

**Objective:** The aim was to validate the General Medication Adherence Scale (GMAS) (English version) in Saudi patients with chronic disease.

**Methods:** A month-long study was conducted in the out-patient department of tertiary care hospitals in three cities of Saudi Arabia that collected data from a randomized sample of Saudi patients with chronic disease. The study aimed to achieve an item-to-subject ratio greater than 1:10. Factor analyses were conducted and fit indices calculated. Convergent, discriminant, known group, and concurrent validities were analysed. Internal consistency was determined using test–retest reliability using Cronbach’s alpha (α), McDonald’s coefficient omega (ω*_t_*), and Pearson’s correlation coefficient (ρ). Sensitivity analysis was conducted. Data were analysed through Statistical Package for Social Sciences (SPSS) version 23. The study was ethically approved (i.e., IRB-129-26/6/1439).

**Results:** The survey gathered responses from 171 patients with a response rate of 85.5%. An item-to-subject ratio of 1:15 was achieved. Factor analysis revealed a three-factor structure with acceptable fit indices (i.e., normed fit index (NFI) = 0.93, Tucker–Lewis index (TLI) = 0.99, and comparative fit index (CFI) = 0.99), i.e., greater than 0.9. The value of root mean square error of approximation (RMSEA) was 0.01, i.e., less than 0.08. The tool established construct validity, i.e., convergent and discriminant validities. Known group and concurrent validities were also established. An α value of 0.74 and ω*_t_* value of 0.92 were reported. Test–retest reliability ρ = 0.82, *p* < 0.001. The tool had high sensitivity (>75%) and specificity (>80%).

**Conclusion:** The GMAS-English was successfully validated in Saudi patients with chronic disease.

## Introduction

Adherence to medication could be defined as the extent of patient concordance to prescribed medication therapy (Osterberg and Blaschke, 2005). It is important in evaluating treatment success as well as identifying drug-related problems (Osterberg and Blaschke, 2005; Naqvi et al., 2018a). Proper adherence ensures optimal disease outcomes and improves patient’s health status, which may not only be limited to clinical status but may incorporate health-related quality of life (Naqvi et al., 2017). This is vital in managing noncommunicable diseases (NCDs) that require long-term medication therapy. NCDs or chronic diseases are continued illnesses that are not completely cured but could be managed effectively by medication therapy (Sabaté, 2003; Dowrick et al., 2005).

According to the World Health Organization (WHO), an estimated 78% of total deaths in Saudi Arabia are related to NCDs. Cardiovascular diseases account for the highest mortality proportion, i.e., 48% and a death probability of 17% between age 30 and 70 years (World Health Organization, 2014). Data from the Institute for Health Metrics and Evaluation reports ischaemic heart disease (IHD) as the most common cause of death followed by cerebrovascular illnesses and chronic kidney disease. Pulmonary diseases, most commonly asthma and to some extent chronic obstructive pulmonary disease, are also included in the list of major causes of death (Institute of Health Metrics and Evaluation, 2017). Endocrine disorder, namely, diabetes mellitus (DM), is another major cause of death in Saudi patients. Other chronic illnesses include Alzheimer’s disease and liver cancer (World Health Organization, 2014). Musculoskeletal problems are the most common cause of disability in Saudi patients; however, IHD is the most frequent cause of disability and death combined. Most common risk factors that precipitate premature death and disability together are high body mass index, high blood pressure, and dyslipidaemia (Institute of Health Metrics and Evaluation, 2017).

Since three quarters of deaths and most disabilities in the Saudi population occur due to chronic diseases, it is imperative to manage them with appropriate medication therapy. An easy approach to appraise treatment success is the use of self-reported medication adherence questionnaire. Only Morisky’s Medication Adherence Scale (MMAS) had been validated in a Saudi population (AlHewiti, 2014; Shilbayeh et al., 2018). However, the tool does not measure cost-related non-adherence, which is an important aspect in adhering to treatment in a developing country like Saudi Arabia (Hussain et al., 2014; Naqvi et al., 2016; Naqvi et al., 2018b). Henceforth, there was a need to validate an instrument that incorporates this aspect in measuring adherence to medication.

We opted for the General Medication Adherence Scale (GMAS) that originated from a developing country. It was developed in Urdu language and was validated. The tool was later translated to English language and subsequently validated (Naqvi et al., 2018a; Naqvi et al., 2019a). The purpose of this study was to validate the English version of GMAS in educated Saudi patients with chronic disease.

## Methods

A month-long study (April 2018) was conducted in the out-patient department (OPD) of tertiary care hospitals in cities of Khobar, Makkah, and Madinah.

### Target Population and Eligibility Criteria

The study targeted out-patients who suffered from any chronic disease. Male and female patients of Saudi Arabian origin who were able to read and understand English language were invited. Further eligibility criteria included patients aged above 18 years, with or without co-morbidities, diagnosed with a chronic disease at least 3 months before the study, and had a valid prescription for medicines indicated for chronic illness were eligible to participate in the study. Medical information of patients was checked after they consented to participate. This was done to screen patients on the basis of eligibility criteria. Patients who fulfil the criteria were included, while those who did not were excluded. Non-Saudi patients, in-patients, or those who had acute illness, follow-up for surgery, or a surgery planned were left out. Patients assisted by caregivers were excluded on the basis of understanding that their medication needs were being taken care of by caregivers and, therefore, adherence measurement for such patients would not be representative. Patients who did not consent to participate were left out. 

### Patient Recruitment and Randomization

The study was conducted in the OPD of tertiary care hospitals during evening hours from 3 pm to 9 pm on weekends, i.e., Thursday–Saturday. The selection of these timings was based on peak patient OPD visiting hours. Patient recruitment strategy of Naqvi and colleagues were followed (Naqvi et al., 2018a); i.e., randomization was carried out on the basis of a computer-generated list containing medical record number (MRN) of patients scheduled to visit the out-patient clinic on the same day. Patients with MRN ending with an odd number were invited. This sequence was altered every day, i.e., inviting patients with MRN ending with an even number the next day.

### Sample Size Calculation

The sample size calculation was based on statistical aspect. Therefore, we calculated sample size on the basis of item-to-subject ratio. Available literature suggests a ratio of 1:5 up to 1:10 (Osborne and Costello, 2004; Dowrick et al., 2005). Therefore, the required sample size was *N* = 55 to 110. However, we gathered data from 171 patients, which increased the ratio to 1:15. Hence, our sample size was more than the required number for tool validation studies (Williams and Brown, 2010).

### Research Instrument

The GMAS tool was initially developed by Naqvi and colleagues in Urdu language for Pakistani patients (Naqvi et al., 2018a). It was subsequently translated in English language and validated in educated Pakistani patients (Naqvi et al., 2019a). We used the English version of GMAS zin this population. Permission to use the tool was obtained. The GMAS was provided to patients in the form of a self-administered tool.

### Factor Analyses

The validation process consisted of exploratory factor analysis (EFA) and partial confirmatory factor analysis (PCFA). Incremental fit indices, namely, normed fit index (NFI), comparative fit index (CFI), and Tucker–Lewis index (TLI), were calculated. A value of NFI, CFI, and TLI greater than 0.9 highlighted good model fit (Zwick and Velicer, 1986). Besides, absolute fit indices such as root mean square error of approximation (RMSEA) were also calculated. A value of RMSEA less than 0.08 demonstrated a good model fit (Pett et al., 2003; Hair et al., 2009; Shima et al., 2015).

### Convergent and Discriminant Validities

The convergent validity of GMAS was established if the average factor loading on a component was greater than 0.7. Moreover, discriminant validity was established if the squared correlation coefficient (ρ^2^) obtained between two constructs was less than their average variance. Both convergent and discriminant validities constitute construct validity of a psychometric tool (Cronbach and Meehl, 1955).

### Known Group Validity

The study hypothesized that adherence to medication would decrease in patients with more co-morbidities and medicines prescribed. Further, adherence is likely to be higher in patients with higher income. This was established by correlating individual adherence score obtained from the second construct of GMAS, i.e., non-adherence due to additional disease and pill burden, with number of medicines prescribed, and number of co-morbidities. In addition, the adherence score obtained from the third construct of GMAS; i.e., cost-related non-adherence was correlated with monthly family income of patients. Pearson’s correlation was used to test the relationship, and values obtained were indicated by a correlation coefficient (ρ). A value of ρ greater than 0.75 and *p*-value less than 0.05 was considered significant (Cohen, 1988; De Vellis, 1991; Kurlander et al., 2009; Iuga and McGuire, 2014).

### Concurrent Validity

The concurrent validity of GMAS was established by correlating the overall adherence score with patient compliance to therapy for a duration of 4 weeks that was based on pill count (Farmer, 1999; Lam and Fresco, 2015). Compliance was calculated as a percentage using the formula below (Naqvi et al., 2019a).

$$Compliance=\frac{Number\;of\;doses\;taken\;by\;patient}{Number\;of\;doses\;prescribed\;to\;patient}\times 100\%$$

Pearson’s correlation was used to test the relationship, and values obtained were indicated by a correlation coefficient (ρ). A value of ρ greater than 0.75 in the positive or negative direction and *p*-value less than 0.05 was considered significant (Cohen, 1988; De Vellis, 1991).

### Internal Consistency

Internal consistency was analysed by test–retest method using Cronbach’s alpha (α) values. A value of α equal to 0.5 or above is considered acceptable. In addition, composite reliability using McDonald’s coefficient (ω*t*) was also used to estimate reliability as an alternative (McDonald, 1999; Trizano-Hermosilla and Alvarado, 2016). Pearson’s correlation (ρ) was used to evaluate test–retest reliability at follow-up after 4 weeks. Studies recommend a period of 4 weeks or 30 days for test–retest purpose (Streiner and Norman, 2003; Wang et al., 2013). A value of ρ greater than 0.75 and *p*-value less than 0.05 was considered significant (Cohen, 1988; De Vellis, 1991). Item-to-total correlation (ITC) and intra-class correlation coefficients (ICC) were also calculated. ITC and ICC were considered acceptable if their value was greater than 0.2 (Lahey et al., 1983; Bowling, 2009; Sushil and Verma, 2010). A Cronbach’s alpha (α) value of 0.5 or greater is considered acceptable (De Vellis, 1991; Cohen, 1988). A McDonald’s coefficient value of 0.7 is accepted (McDonald, 1999; Trizano-Hermosilla and Alvarado, 2016).

### Sensitivity and Specificity Analyses

The tool was checked for sensitivity and specificity to screen patients on the basis of their self-reported adherence and actual compliance levels. Moreover, likelihood ratios, predictive values, and accuracy of tool were reported. Percentage (%) and confidence interval ranges were used to report sensitivity, specificity, predictive values, and accuracy. The method used to calculate the confidence interval ranges for sensitivity, specificity, and accuracy was Clopper–Pearson, while log method was used to calculate the same for likelihood ratios (Altman et al., 2000). Standard logit confidence interval is used to report the same for predictive values (Zhou et al., 2002; Mercaldo et al., 2007).

### Ethics Approval

The study was approved by Institutional Review Board, General Directorate of Health Affairs, Ministry of Health, Saudi Arabia (IRB-129-26/6/1439). All patients were briefed about the study, and a written informed consent was obtained prior to handing the questionnaire. Only consenting patients were included in the study.

## Results

### Demographic Information

A total of 171 patients responded to the survey out of 200 who were approached, giving a response rate of 85.5%. The mean age of patients was 51 ± 16.7 years. Gender distribution was almost equal as 86 patients (50.3%) were males and remaining (*N* = 85, 49.7%) were females. Three quarters of patients (*N* = 127, 74.3%) indicated that they were married, and slightly less than half of patients (*N* = 74, 43.3%) were graduates. A similar proportion (*N* = 73, 42.7%) had co-morbidity (**Table 1**).

**Table 1:** Participants’ information.

	Participants’ information	Sample (*N*)	Percentage
**1.**	**Gender**		
	Male	86	50.3
	Female	85	49.7
**2.**	**Marital information**		
	Single	30	17.5
	Married	127	74.3
	Other (divorced, widowed)	14	8.2
**3.**	**Education**		
	Primary (up to 6 years)	56	32.7
	Secondary (up to 12 years)	41	24
	Graduate (up to 16 years)	74	43.3
**4.**	**Co-morbidity**		
	Yes	73	42.7
	No	98	57.3
**5.**	**Monthly family income**		
	Between SAR 1,000 to 5,000, i.e., USD 266.7–1,333.3	10	5.8
	Between SAR 5,000 to 10,000, i.e., USD 1,333.3–2,666.6	119	69.6
	More than SAR 10,000, i.e., more than USD 2,666.6	42	24.6

The value of United States dollar (USD) corresponds to the exchange rate of USD to Saudi Arabian Riyal (SAR) at the time of this writing, i.e., 10 April 2019.

### Patient Medical Information

Most patients suffered from DM (*N* = 53, 31%), hypertension (*N* = 51, 29.8%), asthma (*N* = 14, 8.2%), and various musculoskeletal disorders (i.e., osteoporosis, osteoarthritis, gout, and rheumatoid arthritis, *N* = 13, 7.6%). Seven patients (4.1%) suffered from thyroid disorders, i.e., hyperthyroidism and hypothyroidism, and another seven (4.1%) had dyslipidaemia. Five patients (2.9%) appeared to have central nervous system disorders [namely, Parkinson’s disease, attention-deficit hyperactivity disorder (ADHD), epilepsy, etc.]. In addition, five patients (2.9%) had sickle cell disease. Four patients (2.3%) had IHD, and four (2.3%) had other illnesses, namely, glaucoma, renal failure, deep vein thrombosis (DVT), etc.

With regard to co-morbidity, the majority of patients (*N* = 73, 42.7%) had one to two co-morbidities and indicated endocrine disorders (*N* = 36, 21.1%), followed by patients (*N* = 20, 11.7%) who had cardiovascular disorders as a co-morbidity. More than half of patients (113, 61.1%) had between one and two medicines prescribed (**Table 2**).

**Table 2:** Disease characteristics of participants.

	Disease characteristics	Sample (*N*)	Percentage
**1.**	**Chronic illness of patients**		
	Diabetes mellitus (DM)	53	31
	Hypertension (HTN)	51	29.8
	Asthma	14	8.2
	Musculoskeletal diseases [osteoporosis (OP), osteoarthritis (OA), gout, and rheumatoid arthritis (RA)]	13	7.6
	Gastrointestinal diseases [irritable bowel syndrome (IBS), Crohn’s disease, gastrointestinal reflex disorder (GERD), ulcerative colitis]	8	4.7
	Thyroid disorders (hyperthyroidism and hypothyroidism)	7	4.1
	Dyslipidaemia	7	4.1
	CNS disorders [Parkinson’s, attention-deficit hyperactivity disorder (ADHD), epilepsy]	5	2.9
	Sickle cell anaemia (SCA)	5	2.9
	Ischaemic heart disease (IHD)	4	2.3
	Other illnesses [glaucoma, renal failure, deep vein thrombosis (DVT), varicose veins]	4	2.3
**2.**	**Type of co-morbidity of patients**		
	No co-morbidity (not applicable)	98	57.3
	Endocrine (DM, dyslipidaemia, thyroid disorders)	36	21.1
	Cardiovascular (HTN, IHD, angina pectoris, DVT)	20	11.7
	Musculoskeletal (OA, OP, RA, and gout)	5	2.9
	Gastrointestinal disease [peptic ulcer disease (PUD), GERD]	4	2.3
	Central nervous system (CNS) diseases (Parkinson’s, epilepsy)	3	1.8
	Renal disease	2	1.2
	Pulmonary disease (asthma, chronic obstructive pulmonary disorder, etc.)	2	1.2
	Skin disease	1	0.5
**3.**	**Number of medicines prescribed**		
	Between 1 and 2 medicines	113	66.1
	Between 3 and 5 medicines	47	27.5
	More than 5 medicines	11	6.4

### Patient Medication Adherence Scores

The mean score for patient behaviour-related non-adherence was reported at 10.4 ± 3.2, mean score for additional disease and pill burden was reported at 9.7 ± 2.3, and mean score for cost-related non-adherence was reported at 4.4 ± 1.3. The mean overall adherence score was reported at 24.6 ± 5.2. Most patients (*N* = 85, 49.7%) were partially adherent to their medication therapy (**Table 3**).

**Table 3:** Adherence scores.

GMAS score	High		Good		Partial		Low		Poor	
	N	%	N	%	N	%	N	%	N	%
Patient behaviour related	58	33.9	29	17	52	30.4	24	14	8	4.7
Additional disease and pill burden	84	49.1	45	26.3	34	19.9	6	3.5	2	1.2
Cost-related non-adherence	42	24.6	47	27.5	72	42.1	8	4.7	2	1.2
Overall adherence	30	17.5	43	25.1	85	49.7	10	5.8	3	1.8

### Factor Analyses

The factor structure was analysed through EFA using principal component analysis (PCA) with varimax rotation. A Kaiser–Meyer–Olkin (KMO) measure of sampling adequacy was obtained at 0.705 and significant (*p*-value < 0.0001). Bartlett’s test of sphericity has χ^2^ value of 370.429. EFA extracted three factors with eigenvalue greater than 1.0. The average variance obtained was 62%. Factor loadings greater than 0.3 on a component and non-salient loading less than 0.3 on others were considered as a single factor. The first five items were loaded on factor 1, the next four on factor 2, and last two on factor 3 (**Table 4**).

**Table 4:** Factor structure.

Constructs	Items	Components		
		1	2	3
1	1	0.742		
	2	0.767		
	3	0.742		
	4	0.653		
	5	0.840		
2	6		0.662	
	7		0.695	
	8		0.776	
	9		0.653	
3	10			0.738
	11			0.693

The EFA was followed by PCFA using maximum likelihood with varimax rotation. The number of factors fixed was at three. The goodness-of-fit test revealed a χ^2^ value of 55.462 (df = 55). All non-salient factor loadings were normally distributed with a mean score of 0.2. The value for NFI was reported at 0.93, CFI was reported at 0.99, and TLI was reported at 0.99, i.e., greater than 0.9. The RMSEA was reported at 0.01, i.e., less than 0.08. These results confirmed a good model fit.

### Convergent and Discriminant Validities

Convergent validity for a construct was established if the average factor loading obtained was above 0.7. The average factor loadings obtained for the first, second, and third constructs were 0.75, 0.7, and 0.72, respectively, which established convergent validity of all three constructs. The average variance between the first and second constructs was 0.508, and squared correlation coefficient (ρ^2^) was 0.258. Similarly, the average variance between the first and third constructs was 0.599, and ρ^2 ^was 0.097. The average variance between the second and third constructs were 0.626, and ρ^2 ^was 0.09. Hence, discriminant validity was established for all three constructs. Both convergent and discriminant validities confirmed the construct validity of the tool.

### Known Group Validity

**Figure 1:**
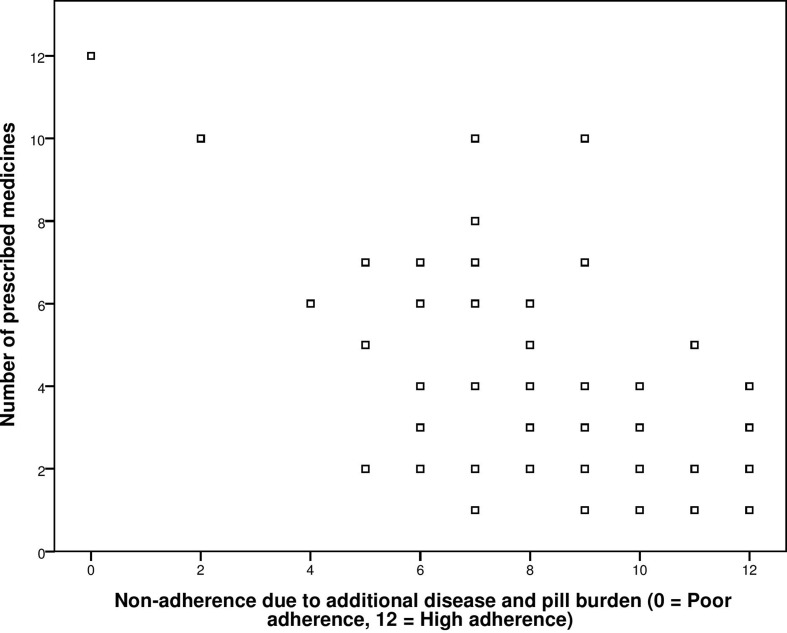
Correlation between General Medication Adherence Scale (GMAS) construct (additional disease and pill burden) and number of prescribed medicines.

The known group validity of GMAS was checked by correlating the adherence score obtained from the second construct of scale, i.e., additional disease and pill burden, with number of co-morbidities and number of medicines. The value for Pearson’s correlation coefficient (ρ) obtained from correlation between adherence score and number of medicines was −0.751 with significant *p*-value < 0.001 (**Figure 1**).

**Figure 2:**
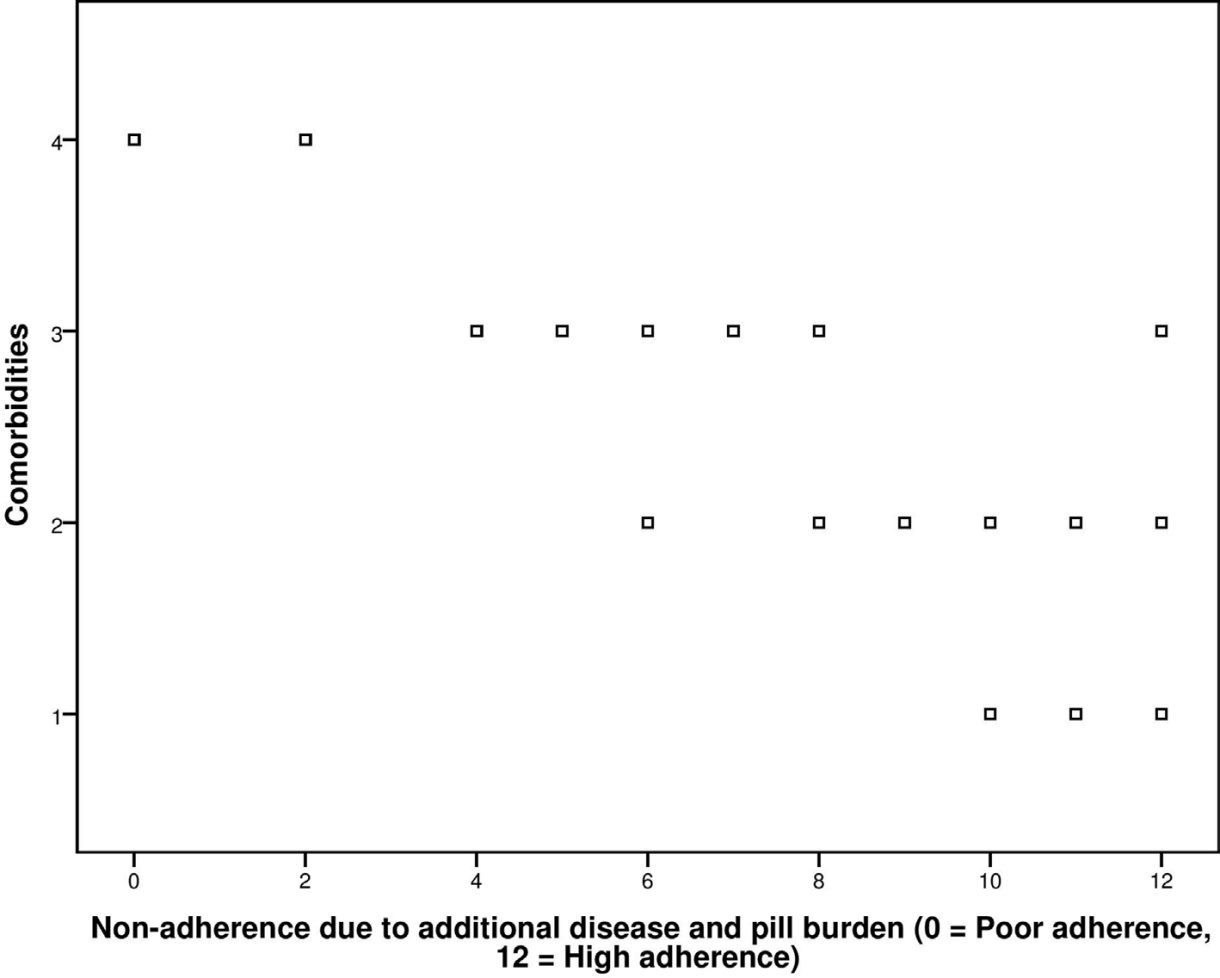
Correlation between GMAS construct (additional disease and pill burden) and co-morbidities.

The correlation coefficient obtained from correlation between adherence score and co-morbidities was −0.840 with significant *p*-value < 0.001 (**Figure 2**).

**Figure 3:**
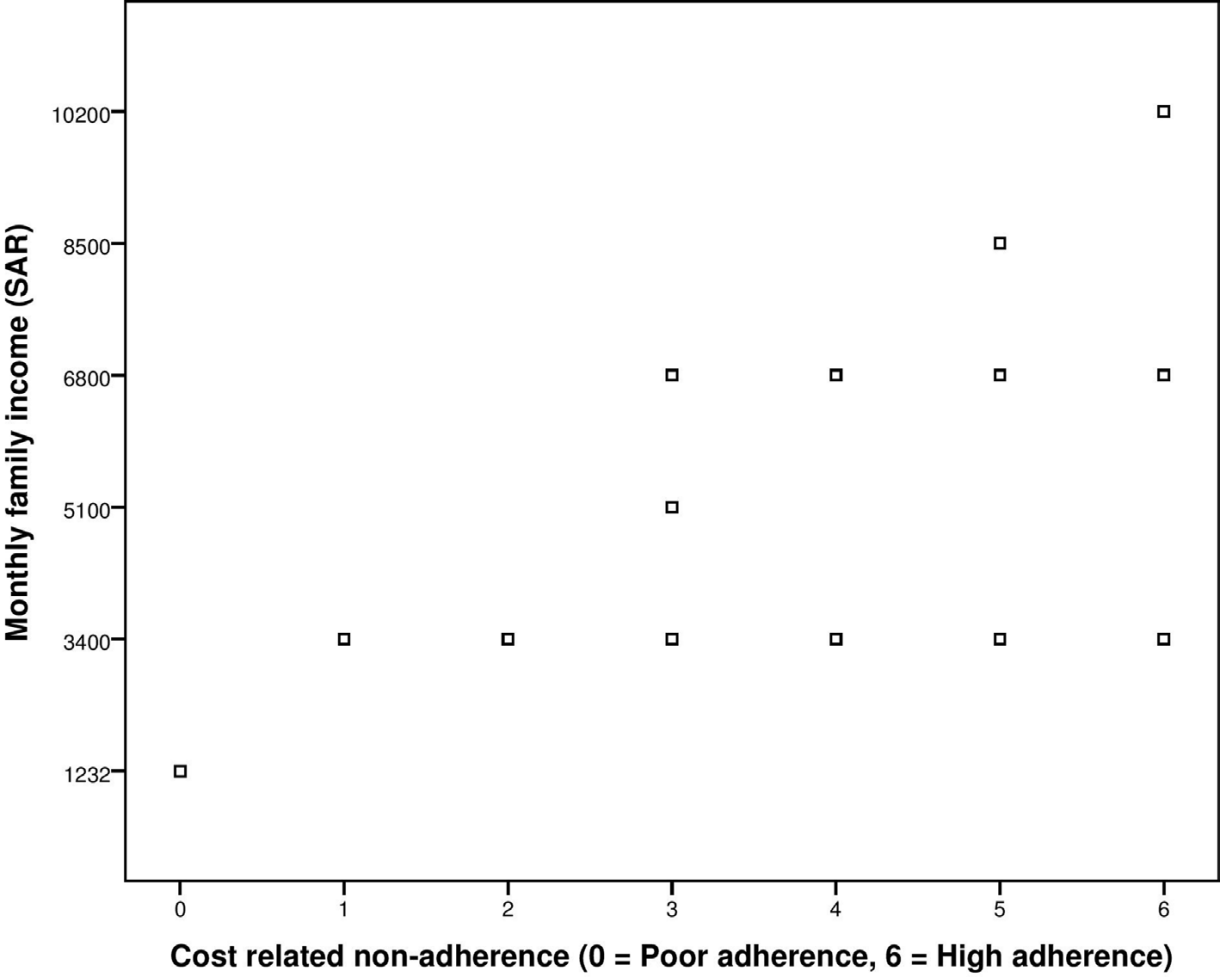
Correlation between GMAS construct (cost-related non-adherence) and monthly family income.

Additionally, correlation of adherence score obtained from the third construct of scale, i.e., cost-related non-adherence, with the demographic variable of monthly family income was also conducted. The ρ was high, i.e., 0.794, and significant at *p*-value of less than 0.001 (**Figure 3**).

### Concurrent Validity

**Figure 4:**
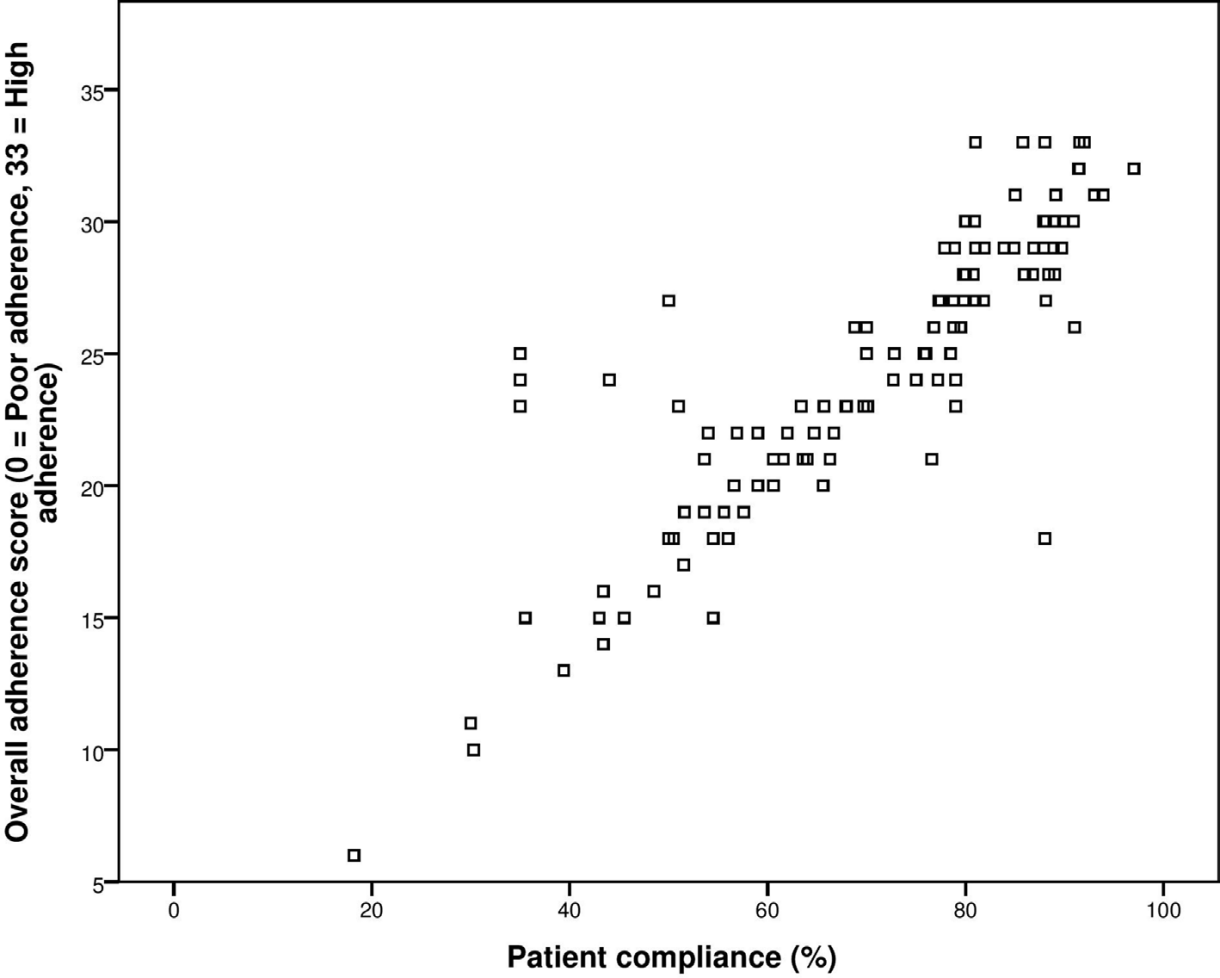
Correlation between overall adherence score and (%) compliance to medication therapy.

The concurrent validity was established by correlating the self-reported adherence score of patients with their actual compliance to medication therapy after 4 weeks. The test–retest Pearson’s correlation was 0.883 with significant *p*-value of less than 0.001. Hence, concurrent validity was established (**Figure 4**).

### Internal Consistency

Reliability analysis reported a Cronbach’s alpha (α) value of 0.74 for 11 items. The test–retest reliability coefficient (ρ) was reported at 0.82, *p*-value < 0.001. All items appeared to positively correlate with each other except for item 10, which was negatively correlated with items 5, 7, and 11. The composite reliability analysis reported a McDonald’s coefficient omega (ω*t*) of 0.92 for 11 items. Analyses of individual constructs reported an α value of 0.708 and ω*t* value of 0.82, for the first construct that contained five items. All items appeared to positively correlate with each other with minimum correlation coefficient value > 0.177; ICC was reported at 0.708 (0.632–0.772 for 95% CI). Besides, the second construct reported an α value of 0.654 and ω*t* value of. 0.72. All items appeared to positively correlate with each other with minimum correlation coefficient value > 0.236; ICC was reported at 0.654 (0.560–0.731 for 95% CI). Furthermore, the third construct reported an α value of 0.232 and ω*t* value of 0.333. All items appeared to positively correlate with each other with minimum correlation coefficient value > 0.104, ICC was reported at 0.232 (0.160–0.666 for 95% CI). 

### Sensitivity Analyses

The sensitivity of GMAS to screen patients with high to good adherence based on actual compliance was 86.67% (75.41%–94.06%), while its specificity was reported at 81.08% (72.55%–87.89%). The positive likelihood ratio was reported at 4.58 (3.08–6.82), while negative likelihood ratio was 0.16 (0.09–0.32). A positive predictive value of 71.23% (62.46%–78.66%) was reported. A negative predictive value of 91.84% (85.43–95.57%) was reported. The accuracy of GMAS was reported at 83.04% (76.56%–88.34%).

## Discussion

Adherence is an important parameter to judge the likelihood of treatment success and one of the cornerstones of chronic disease state management (Sabaté, 2003; Osterberg and Blaschke, 2005; Naqvi et al., 2019b). Several methods are available to document patients’ medication adherence, which include direct methods such as biochemical assays and medication event monitoring systems, as well as indirect methods such as pill counts and self-reported measures (Velligan et al., 2007; Lam and Fresco, 2015). Notwithstanding the quality and accuracy of direct methods, their sophisticated nature and cost have, to some extent, limited their use in common practice (Farmer, 1999; Lam and Fresco, 2015). An indirect method such as self-reported adherence by patients using a questionnaire is one of the most convenient and inexpensive ways of documenting adherence. However, indirect methods have their own limitations such as limited generalizability, long format that may be time-consuming, and difficulty to understand the questions, which may result in overestimation or underestimation of adherence (Farmer, 1999; Nguyen et al., 2014; Tan et al., 2014; Lam and Fresco, 2015).

There is no gold standard to measure patient adherence to medicines (Perez-Escamilla et al., 2015; Forbes et al., 2018). Therefore, studies have recommended a mix of different approaches to measure adherence (Lam and Fresco, 2015). Forbes and colleagues have recommended using a combination of tools to measure adherence to have a value that is deemed closer to reality (Forbes et al., 2018; Naqvi and Hassali, 2018). Lam and Fresco have recommended a multi-measure approach to document adherence by either selecting direct and indirect methods or combining objective and subjective measures within an indirect approach (Lam and Fresco, 2015). Evidence indicates that combination of objective and subjective measure provides highly reliable results (Rapoff, 2010).

We selected the combination of subjective and objective approaches to validate GMAS in Saudi patients. The objective method selected was pill count method, which measured doses taken at two time points. These data were used to measure compliance rate of patients. We selected this objective approach due to its high accuracy as reported in literature (Farmer, 1999; Lam and Fresco, 2015).

The GMAS was originally formulated for developing countries with an insight into documenting patient adherence to medication including determinants such as cost, co-morbidity, and pill burden, which impact adherence. The sample size required for a tool validation study depends upon the number of items in questionnaire. On the basis of item-response theory, the figure that was required to successfully validate the tool was 55–110 patients (De Vellis, 1991; Pett et al., 2003). Nevertheless, study exceeded this figure and collected data from a randomized sample of 171 patients. This sample count was higher than that used by another study that validated the eight-item Morisky’s Medication Adherence Scale (MMAS-8) in a Saudi population (Shilbayeh et al., 2018). This aspect could be regarded as a strength of our study.

Patients with several chronic illnesses such as cardiovascular, endocrine, pulmonary, musculoskeletal, gastrointestinal, central nervous system related, and blood diseases were a part of this study. This sample included patients with all those chronic illnesses that were regarded as major cause of death and disability in Saudi Arabia by international health agencies (World Health Organization, 2014; Institute of Health Metrics and Evaluation, 2017). This highlights its sampling adequacy from health perspective and presents a justification to its generalizability.

Statistical analysis revealed a high sampling adequacy, i.e., KMO greater than 0.7. Factorial analyses revealed a three-factor solution. This finding was in line with previous validation results of the tool in Pakistani patients (Naqvi et al., 2018a; Naqvi et al., 2019a). Both absolute and incremental fit indices were calculated, and the values obtained from the indices provided a strong indication of a good three-factor model fit.

The tool established convergent and discriminant validities for all three constructs. Only MMAS-8 has been validated in a Saudi population as of now (AlHewiti, 2014; Shilbayeh et al., 2018). However, it would not serve as a benchmark since it does not measure cost-related non-adherence. Moreover, it has no sub-domain; therefore, discriminant validity results could not be compared as well. Therefore, the statistical approach was selected, and a threshold value of 0.7, as per literature, was considered a criterion for establishing convergent validity (De Vellis, 1991). Similarly, if the average variance between two constructs was greater than squared correlation coefficient, discriminant validity was established (De Vellis, 1991). This approach has also been used by Naqvi et al., (2018a) and Naqvi et al., (2019a) in previous studies involving GMAS. 

Reliability analysis reported an alpha value of 0.74, which highlighted a strong internal consistency of the tool, i.e., greater than 0.5, and was equal or greater than the values reported by previous studies that used MMAS-8 in Saudi patients (AlHewiti, 2014; Mayet, 2016; Shilbayeh et al., 2018). It was higher than the value reported from Arabic version of MMAS-8 (Ashur et al., 2015). In addition to this, we estimated reliability through composite reliability using McDonald’s coefficient omega (ω*_t_*). It is a newer technique to estimate reliability and is regarded as a better alternative to Cronbach’s alpha (α). This method also considers the strength of associations between items and item-specific measurement errors (Lucke, 2005; Graham, 2006). The reliability estimate provided by omega (ω*_t_*) is more realistic and closer than alpha (α) (McDonald, 1999; Trizano-Hermosilla and Alvarado, 2016). The GMAS is the only psychometric adherence measuring tool used that is deemed reliable using McDonald’s coefficient (ω*_t_*) and currently the only tool that estimated reliability using both techniques.

Moreover, the GMAS established known group and concurrent validities. A previous study by Shilbayeh and colleagues using MMAS-8 could not establish its concurrent validity. Furthermore, the sensitivity of GMAS was greater than 85%, while its specificity was above 80%. A previous study using Arabic version of MMAS-8 reported sensitivity of 55.7% and specificity of 50% (Mayet, 2016). Hence, these aspects could be regarded as other strengths of GMAS in a Saudi population.

The successful validation of GMAS-English in this population may serve as a step towards its Arabic translation and subsequent validation in a Saudi population. Since Arabic is the national language of most countries in the region, the Arabic version could be used to measure adherence in other Arabic-speaking countries. Our target segment included only those Saudi patients who were able to read and understand English language. This is a limitation; however, it would be negligible since we conducted randomization that negated selection bias. The GMAS achieved a response rate above 80% in Saudi patients.

## Conclusion

The tool demonstrated adequate internal consistency and established convergent, discriminant, known group, and concurrent validities. Factor analysis results corresponded to the hypothetic constructs. All values relating to validation aspects were higher than those obtained from other tools previously. The GMAS-English was deemed a validated tool to measure medication adherence in Saudi patients suffering from chronic illness. The authors recommend translating this tool into Arabic language and validating it in this population.

## Ethics Statement

The study was approved by Institutional Review Board, General Directorate of Health Affairs, Ministry of Health, Saudi Arabia (IRB-129-26/6/1439).

## Author Contributions

The idea was jointly conceived by AN and MH, and both authors designed the study. AN and MM collected the data with AH, SG, HF, and DA-S. SG entered the data in SPSS. AN, DA, AH, HF, MM, and MH analysed the data. DA and MM assisted in ethical approval process and editing of the manuscript. All authors were equally involved in revising the manuscript during the peer review process. All authors read and approved the final version of the manuscript.

## Conflict of Interest Statement

The authors declare that the research was conducted in the absence of any commercial or financial relationships that could be construed as a potential conflict of interest.
